# Serial high-sensitivity cardiac troponin I trajectories predict cardiovascular events in hemodialysis patients

**DOI:** 10.1093/ckj/sfaf294

**Published:** 2025-09-22

**Authors:** Yue Wu, Fengqin Li, Qiao Fu, Yanzhe Wang, Jialing Wang, Xinyue Chen, Tong Wu, Qijie Chen, Nan Zhang, Dingyu Zhu, Xiaoxia Wang

**Affiliations:** Department of Nephrology, Shanghai Tongren Hospital, Shanghai Jiao Tong University School of Medicine, Shanghai, China; Department of Nephrology, Shanghai Tongren Hospital, Shanghai Jiao Tong University School of Medicine, Shanghai, China; Department of Nephrology, Shanghai Tongren Hospital, Shanghai Jiao Tong University School of Medicine, Shanghai, China; Department of Nephrology, Shanghai Tongren Hospital, Shanghai Jiao Tong University School of Medicine, Shanghai, China; Department of Nephrology, Shanghai Tongren Hospital, Shanghai Jiao Tong University School of Medicine, Shanghai, China; Department of Nephrology, Shanghai Tongren Hospital, Shanghai Jiao Tong University School of Medicine, Shanghai, China; Department of Nephrology, Shanghai Tongren Hospital, Shanghai Jiao Tong University School of Medicine, Shanghai, China; Department of Nephrology, Shanghai Tongren Hospital, Shanghai Jiao Tong University School of Medicine, Shanghai, China; Department of Nephrology, Shanghai Tongren Hospital, Shanghai Jiao Tong University School of Medicine, Shanghai, China; Department of Nephrology, Shanghai Tongren Hospital, Shanghai Jiao Tong University School of Medicine, Shanghai, China; Department of Nephrology, Shanghai Tongren Hospital, Shanghai Jiao Tong University School of Medicine, Shanghai, China

**Keywords:** high-sensitivity cardiac troponin I, maintenance hemodialysis, major adverse cardiovascular events

## Abstract

**Background:**

Although commonly elevated in maintenance hemodialysis (MHD) patients without apparent myocardial ischemia, the prognostic significance of serial high-sensitivity cardiac troponin I (hs-cTnI) changes remains underexplored. We aimed to evaluate the association between longitudinal hs-cTnI trajectories and cardiovascular events in MHD patients.

**Methods:**

This retrospective cohort study enrolled 265 clinically stable MHD patients with repeated hs-cTnI measurements (mean six per patient) and followed them until the first major adverse cardiovascular event (MACE) or January 2024. Nonlinear relationships were analyzed using restricted cubic splines, with inflection points identified to assess threshold and saturation effects. Multivariate regression analyses, time-dependent Cox and splines for time were used to investigate the association of hs-cTnI with MACE using single time point (at baseline) and repeated measurements, respectively.

**Results:**

Among 265 MHD patients, the incidence of MACE was 41.9%. Nonlinear relationships between hs-cTnI and MACE were identified, with an inflection point at 64 ng/L. Multiple regression equations showed a 2.06-fold increased risk of MACE in the high baseline hs-cTnI group compared with the low baseline hs-cTnI group after adjusting for numerous influencing factors. After full adjustment, each 10 ng/L increase in the time-updated hs-cTnI value remained significantly associated with a 2.71% elevated risk of MACE. Splines for time demonstrated that patients with MACE exhibited higher hs-cTnI concentrations and steeper trajectories compared with those without MACE.

**Conclusion:**

Serial hs-cTnI changes independently predict cardiovascular risk in MHD patients, suggesting that dynamic measurement of hs-cTnI may be useful for monitoring cardiovascular prognosis and early intervention in this high-risk population.

## GRAPHICAL ABSTRACT

**GRAPHICAL ABSTRACT:**
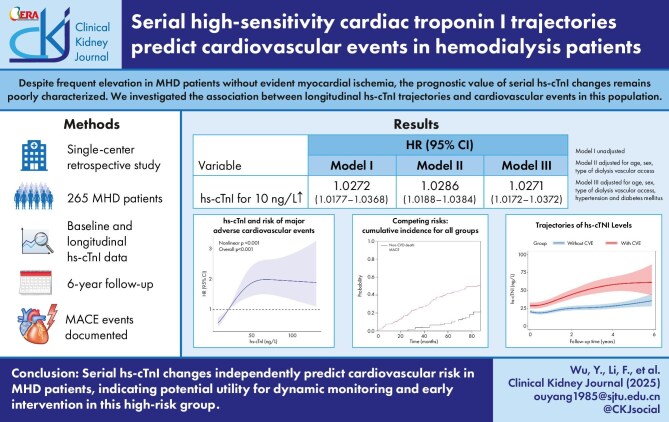

KEY LEARNING POINTS **What was known:**

- Elevated high-sensitivity cardiac troponin I (hs-cTnI) is common in maintenance hemodialysis (MHD) patients, but its prognostic value for cardiovascular events remains unclear.
- Previous single-time-point studies lacked evidence on serial changes.
- This study examines whether longitudinal hs-cTnI trajectories improve risk stratification in MHD patients.

**This study adds:**

- Serial hs-cTnI changes independently predict MACE.
- Patients with MACE had higher baseline hs-cTnI and steeper trajectories.
- Dynamic monitoring may improve early risk detection and guide interventions in MHD patients.

**Potential impact:**

- Routine serial hs-cTnI testing could be integrated into cardiovascular risk monitoring for MHD patients.
- Clinicians may use hs-cTnI trajectories to identify high-risk patients for closer follow-up or preventive therapies.

## INTRODUCTION

Cardiovascular disease accounts for approximately 50% of deaths in the dialysis population. While cardiovascular mortality has declined in the general population, this trend has not been observed in dialysis patients [1, 2]. The incidence of cardiovascular events is significantly higher in maintenance dialysis patients compared with the general population [3], with a mortality rate 10–20 times greater [4, 5]. Cardiac troponin (cTn), the most successful cardiac-specific circulating biomarker, is superior to other diagnostic markers for acute myocardial ischemia when measured by fully automated assays [6–8]. Both cTnI and cTnT are preferred biomarkers for identifying acute coronary syndromes [9], with high-sensitivity assays (hs-cTn) recommended by the Fourth Global Unified Definition of Myocardial Infarction [10]. Studies suggest that cTnI levels are more stable post-hemodialysis than cTnT, with greater specificity for myocardial injury in chronic kidney disease (CKD) patients [11–14].

Chronically elevated troponin levels are common in maintenance hemodialysis (MHD) patients without acute myocardial infarction or coronary artery disease. These elevations may reflect chronic myocardial injury rather than epicardial coronary disease [15–20], influenced by factors such as gender, age, neuromuscular disease, blood sampling timing and assay methods [21]. Potential pathophysiological mechanisms include hemodialysis-induced myocardial stress [22], reduced renal clearance [23] and circulatory stasis [24], among others. While hs-cTnI is strongly associated with cardiovascular event risk in the general population [25–28], and temporal increases in hs-cTnI predict elevated risk [29–32], the relationship between hs-cTnI dynamics and cardiovascular events in MHD patients remains underexplored. Therefore, this retrospective cohort study aimed to explore the predictive value of elevated serum hs-cTnI for the occurrence of cardiovascular events in patients with MHD and to analyze the relationship between temporal changes in hs-cTnI and prognosis.

## MATERIALS AND METHODS

### Study population

**Figure 1:**
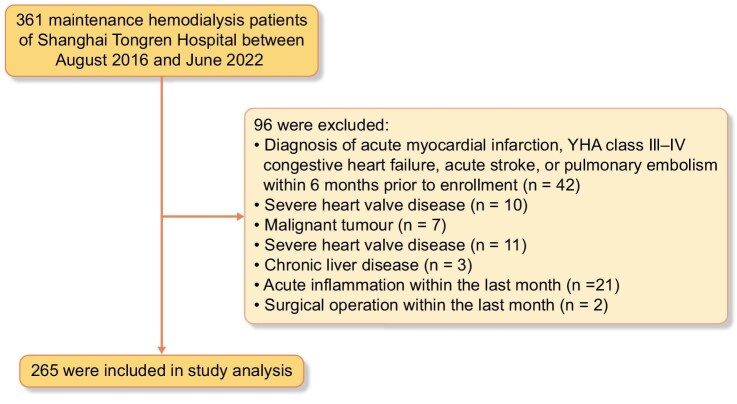
Study population flowchart.

Patients undergoing MHD at the hemodialysis center of Shanghai Tongren Hospital between August 2016 and June 2022 were included in this study. Inclusion criteria were: (i) MHD; (ii) age ≥18 years old; (iii) regular dialysis schedule of three sessions per week, with each session lasting at least 4 h; and (iv) provision of signed informed consent. Exclusion criteria included: (i) diagnosis of acute myocardial infarction, New York Heart Association class III–IV congestive heart failure, acute stroke or pulmonary embolism within 6 months prior to enrollment (*n* = 42); (ii) severe heart valve disease (*n* = 10); (iii) malignant tumor (*n* = 7); (iv) severe heart valve disease (*n* = 11); (v) chronic liver disease (*n* = 3); (vi) acute inflammation within the last month (*n* = 21); and (vii) surgical operation within the last month (*n* = 2). Finally, a total of 265 eligible patients were included (Fig. 1). The study protocol was reviewed and approved by the Ethics Committee of the Tongren Hospital Affiliated to Shanghai Jiao Tong University (Approval No. 2022-095-01). Written informed consent was obtained from participants to participate in the study.

### Observation indicators

(i) Clinical data: gender, age, height, weight, body mass index (BMI), duration on dialysis, underlying renal disease and comorbidities (e.g. diabetes, hypertension).
(ii) Laboratory data: hs-cTnI, creatine kinase isoenzyme, B-type natriuretic peptide (BNP), routine biochemical parameters, complete blood count, lipid profile, parathyroid hormone and left ventricular ejection fraction at baseline and during follow-up.
(iii) hs-cTnI detection method: serum hs-cTnI levels in this study were measured during the biannual routine follow-up laboratory tests performed in patients on MHD. Fasting venous blood samples were collected before dialysis, placed in EDTA anticoagulation tubes, centrifuged, serum separated and stored at –80°C. Hs-cTnI levels were measured using electrochemiluminescence immunoassay, with each patient assessed at least twice at different time intervals during follow-up. Patients were stratified into two groups based on hs-cTnI inflection points identified through threshold and saturation effect analysis.

### Follow-up and endpoint event recording

Major adverse cardiovascular events (MACE) were the primary outcome, defined as cardiovascular death, nonfatal myocardial infarction, coronary artery revascularization, coronary artery disease or stroke (hemorrhagic and ischemic) confirmed by imaging. The cause of death and nature of the first MACE (fatal or nonfatal) were determined by treating physicians blinded to baseline laboratory data. Follow-up data were collected by blinded researchers via telephone interviews, chart reviews, and queries to the Social Security Death Index and records.

### Statistical methods

Data analysis was performed using R (The R Foundation; version 4.2.0) and EmpowerStats software (www.empowerstats.com, X&Y solutions, Inc., Boston, MA, USA). Normality of continuous variables was assessed; normally distributed data were expressed as mean ± standard deviation and compared using *t*-tests, while non-normally distributed data were expressed as median and compared using the Mann–Whitney test. Categorical variables were summarized as frequencies and percentages, with comparisons made using the chi-square test.

Restricted cubic splines and smoothing functions were used to evaluate potential non-linear relationships between variables and to visualize the association between baseline hs-cTnI and MACE. Threshold and saturation effect analyses identified inflection points in baseline hs-cTnI levels using segmented regression. Univariate analyses assessed the impact of other variables on MACE, while multiple regression models explored the independent association of baseline hs-cTnI with MACE after adjusting for confounders. Time-dependent Cox proportional hazards models were used to assess the association between dynamically changing hs-cTnI levels and the risk of MACE. Additionally, the impact of temporal variations in BNP, hemoglobin levels and dialysis vintage—all analyzed as time-dependent covariates—on MACE risk was assessed. Cumulative incidence function (CIF) curves were used to compare the incidence of different outcome events.

Subgroup analyses were conducted to examine the association between hs-cTnI and MACE risk across age, BMI, gender and hypertension subgroups, with interaction analyses performed to assess potential effect modifications. Splines for time were used to visualize trends in hs-cTnI concentrations in patients with and without MACE. Statistical significance was set at *P *< .05 for all analyses.

## RESULTS

### Restricted cubic spline regression analysis

**Figure 2:**
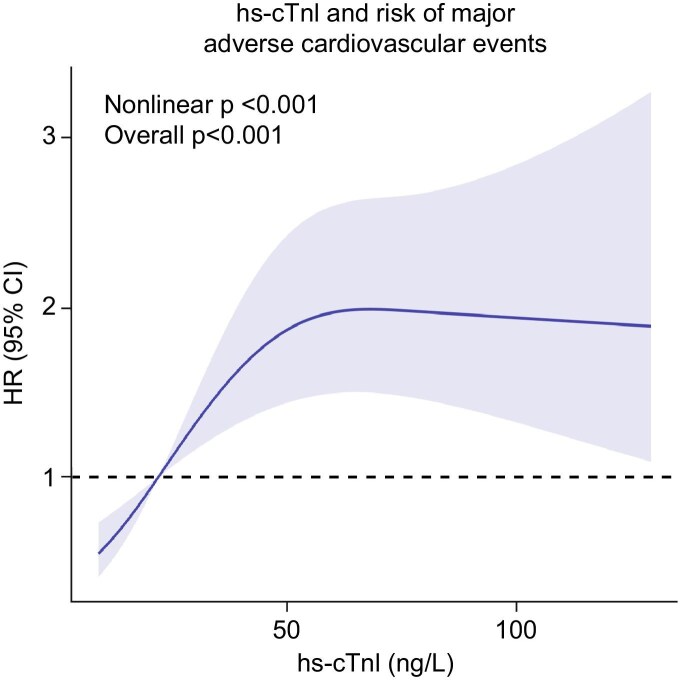
Restricted cubic spline regression analysis demonstrating the association between baseline hs-cTnI levels and the risk of MACE. Adjusted for age, sex, BMI, BNP, hemoglobin, history of hypertension and diabetes mellitus.

Restricted cubic spline regression analysis revealed a significant non-linear relationship between baseline hs-cTnI and the hazard ratio (HR) for MACE (Fig. 2). After adjusting for age, sex and BMI, the non-linear *P*-value was <.001, supporting the use of a non-linear model to describe this association.

### Smooth curve fitting and analysis of threshold saturation effects

**Figure 3:**
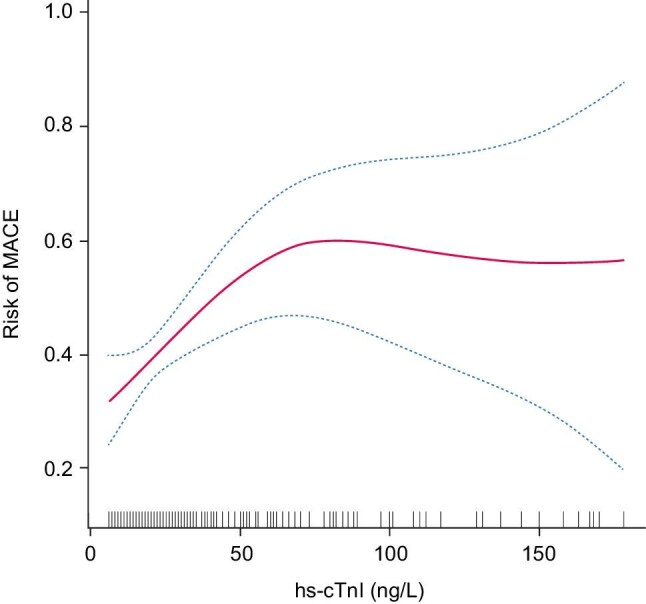
Association between baseline hs-cTnI and risk of MACE. Adjusted for age, sex, BMI, BNP, hemoglobin, history of hypertension and diabetes mellitus.

Smooth curve fitting (Fig. 3) demonstrated a biphasic relationship between baseline hs-cTnI and MACE risk, with an inflection point identified at 64 ng/L. Below this threshold, each increase baseline in hs-cTnI was associated with a 3% higher risk of MACE [HR 1.03 (1.01, 1.04), *P *= .002]. Above 64 ng/L, no significant association was observed [HR 0.99 (0.98, 1.01), *P *= .318] (Table 1). The log-likelihood ratio test confirmed the inflection point (*P *= .016).

**Table 1:** Threshold effect analysis of hs-cTnI and MACE using piecewise linear regression.

Models	OR (95% CI)	*P*
Model I		
One line effect	1.01 (1.00,1.02)	.005
Model II		
Turning point (K)	64	
hs-cTnI < K	1.03 (1.01,1.04)	.002
hs-cTnI ≥ K	0.99 (0.98,1.01)	.318
*P*-value for logarithm likelihood ratio test		.016

The threshold of hs-cTnI for MACE is 64 ng/L. The data are expressed as a ratio, with the HR [95% confidence interval (CI)] and *P*-values indicated.

Model I is a linear analysis, while Model II is a nonlinear analysis. Adjustments were made for age, sex, BMI, BNP, hemoglobin, hypertension and history of diabetes.

### Clinical characteristics of study population

The study included 265 maintenance hemodialysis patients (63.8% male, mean age 65 ± 13 years, mean BMI 22.7 ± 4.2 kg/m²). Median hs-cTnI was 22 ng/L [interquartile range (IQR) 10–44], median dialysis duration was 10.7 months (IQR 4.6–32.0) and median follow-up was 44.7 months (IQR 27.0–67.0). Hypertension was present in 95.1% of patients, diabetes in 45.3%, and 41.9% experienced MACE. Patients were stratified into low-level (hs-cTnI <64 ng/L) and high-level (hs-cTnI ≥64 ng/L) groups. Baseline characteristics were comparable between groups (Table 2).

**Table 2:** Characteristics of the study population according to hs-cTNI.

	Low (<64 ng/L)	High (≥64 ng/L)	
Characteristics	(*n* = 229)	(*n* = 36)	*P*
Age (years)	64.8 (12.8)	66.3 (13.6)	.520
Sex (female), *n* (%)	83 (36.2)	13 (36.1)	.988
BMI (kg/m^2^)	22.6 (4.1)	23.2 (5.0)	.468
Dialysis duration (months)	10.3 (4.7–32.0)	12.1 (3.8–20.5)	.833
Systolic blood pressure (mmHg)	143 (17)	141 (19)	.659
Albumin (g/L)	36.1 (5.7)	35.7 (6.6)	.707
Blood creatinine (μmol/L)	780.3 (294.5)	771.7 (248.5)	.876
Total cholesterol (mmol/L)	3.8 (1.0)	4.1 (1.3)	.248
Hemoglobin (g/L)	94.2 (23.6)	95.8 (27.8)	.708
BNP (pg/mL)	361.6 (118.0–869.7)	403.3 (211.3–2105.7)	.233
C-reactive protein (mg/L)	6.7 (1.9–26.0)	9.6 (3.4–40.1)	.084
Parathyroid hormone (pg/mL)	23.4 (13.3–34.6)	28.6 (9.4–35.9)	.997
Left ventricular ejection fraction	60.4 (5.7)	57.3 (8.0)	.009
Type of dialysis vascular access, *n* (%)			.011
Autogenous arteriovenous fistula	187 (81.7)	22 (61.1)	
Indwelling central venous catheter	29 (12.7)	8 (22.2)	
Artificial arteriovenous fistula	13 (5.7)	6 (16.7)	
Hypertension (yes), *n* (%)	219 (95.6)	33 (91.7)	.306
Diabetes mellitus (yes), *n* (%)	103 (45.0)	17 (47.2)	0.801

Data are presented as mean (standard deviation), median (IQR) or *n* (%).

### Analysis of factors associated with MACE

Univariate analysis (Table 3) identified female sex [HR 1.68 (1.01, 2.79), *P *= .044], hemoglobin [HR 1.01 (1.00, 1.03), *P *= .010] and Creatine Kinase-Myocardial Band (CK-MB) [HR 1.08 (1.01, 1.16), *P *= .022] as significant predictors of MACE. Age, BMI, systolic blood pressure and other factors showed no significant association.

**Table 3:** Univariate analysis for MACE.

Covariate	Statistics	OR (95% CI)	*P*
Age (years)	65 ± 13	1.01 (0.99, 1.03)	.217
Sex, *n* (%)			
Male	169 (63.77)	Reference	
Female	96 (36.23)	1.68 (1.01, 2.79)	.044
Body mass index (kg/m^2^)	22.68 ± 4.23	0.97 (0.92, 1.03)	.315
Systolic blood pressure (mmHg)	142.37 ± 17.57	1.01 (0.99, 1.02)	.453
Calcium (mmol/L)	2.15 ± 0.29	1.67 (0.70, 3.98)	.250
Urea (mg/dL)	24.47 ± 9.76	0.98 (0.95, 1.01)	.138
Total cholesterol (mmol/L)	3.86 ± 1.07	1.17 (0.91, 1.50)	.213
Triglycerides (mmol/L)	1.53 ± 0.87	1.06 (0.78, 1.44)	.700
White blood cell (10^9^/L)	6.58 ± 2.69	1.07 (0.97, 1.17)	.169
Hemoglobin (g/L)	94.40 ± 24.14	1.01 (1.00, 1.03)	.010
D-Dimer (μg/mL)	2.02 ± 3.14	0.97 (0.88, 1.06)	.475
Creatine Kinase-Myocardial Band (ng/L)	3.34 ± 3.92	1.08 (1.01, 1.16)	.022
Left ventricular ejection fraction	60.02 ± 6.16	0.98 (0.94, 1.02)	.407
Type of dialysis vascular access, *n* (%)			
Autogenous arteriovenous fistula, *n* (%)	209 (78.87)	Reference	
Indwelling central venous catheter, *n* (%)	37 (13.96)	0.76 (0.37, 1.56)	.452
Artificial arteriovenous fistula, *n* (%)	19 (7.17)	0.33 (0.11, 1.04)	.058
Hypertension (yes), *n* (%)			
No	13 (4.91)	Reference	
Yes	252 (95.09)	0.83 (0.27, 2.55)	.749
Diabetes mellitus (yes), *n* (%)			
No	145 (54.72)	Reference	
Yes	120 (45.28)	0.92 (0.57, 1.51)	.752

Data are presented as mean ± standard deviation or *n* (%).

### Multiple regression equations

To investigate the relationship between baseline hs-cTnI and MACE, we performed multiple multiple regression equations. In the unadjusted model, each 10 ng/L increase in baseline hs-cTnI was associated with an 8% higher MACE risk [HR 1.08 (1.04, 1.13), *P *< .001]. This association remained significant in Model I (adjusted for age, sex and BMI) [HR 1.09 (1.05, 1.14), *P *< .001] and Model II (further adjusted for age, sex, BMI, months on dialysis, hemoglobin, CK-MB, BNP, uric acid, total cholesterol, triglycerides, type of dialysis vascular access, hypertension and diabetes mellitus) [HR 1.07 (1.01, 1.13), *P *= .020]. In the three models mentioned above, the high-level hs-cTnI group had a 2.20-fold, 2.41-fold and 2.06-fold higher risk of MACE compared with the low-level group, respectively (Table 4).

**Table 4:** Relationship between hs-cTnI and MACE in different models.

Variable	Crude model [ HR (95% CI), *P*]	Model I [ HR (95% CI), *P*]	Model II [HR (95% CI), *P*]
hs-cTnI for 10 ng/L↑	1.08 (1.04, 1.13), *P *< .001	1.09 (1.05, 1.14), *P *< .001	1.07 (1.01, 1.13), *P = *.020
hs-cTnI (ng/L)			
Low (<64 ng/L)	Reference	Reference	Reference
High (≥64 ng/L)	2.20 (1.34, 3.61), *P = *.002	2.41 (1.45, 3.98), *P *< .001	2.06 (1.09, 3.89), *P = *.025

Model I adjusted for: age, sex and BMI.

Model II adjusted for: age, sex, BMI, months on dialysis, hemoglobin, CK-MB, BNP, uric acid, total cholesterol, triglycerides, type of dialysis vascular access, hypertension and diabetes mellitus.

CI, confidence interval.

### The forest plot for different subgroups

**Figure 4:**
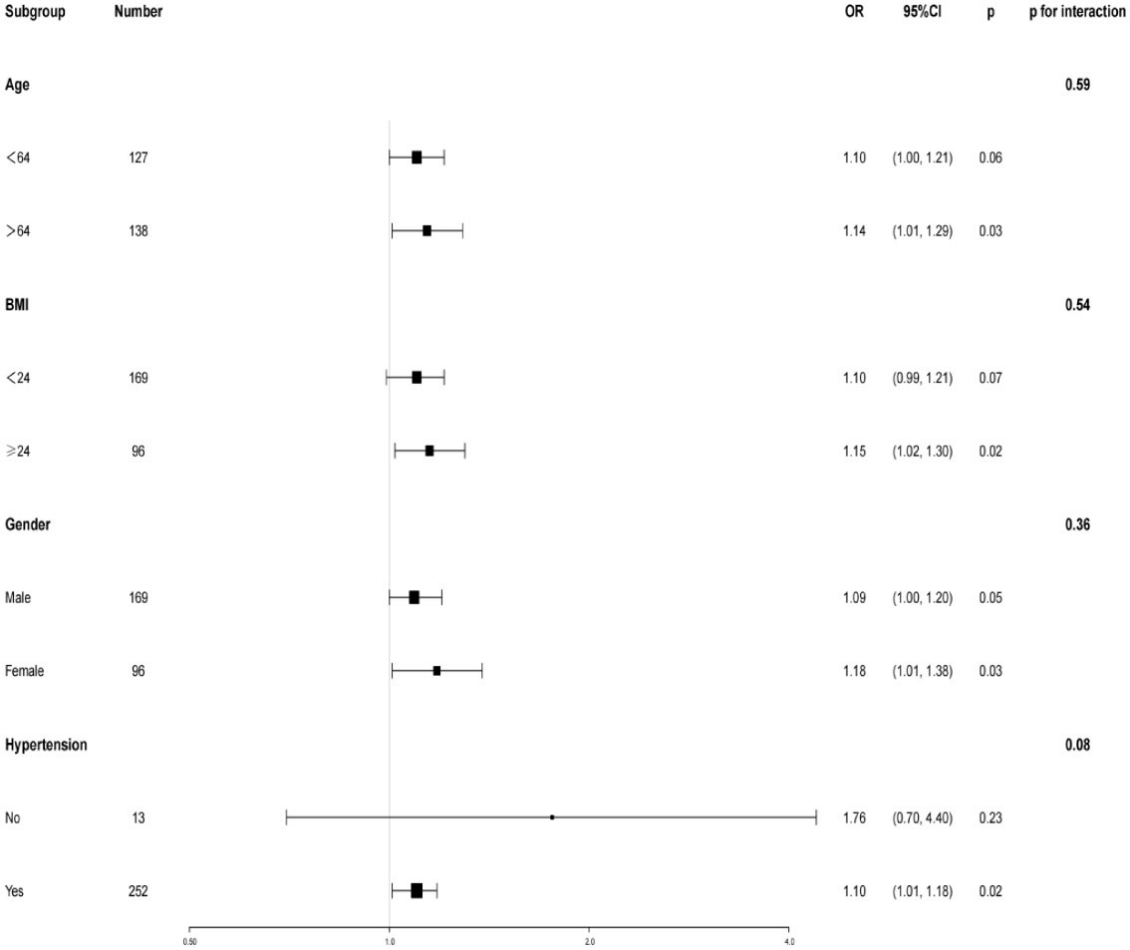
Stratified analysis of the correlation between baseline hs-cTnI and MACE.

Stratified analysis (Fig. 4) revealed consistent associations between baseline hs-cTnI and MACE risk across subgroups. For every 10 ng/L increase in hs-cTnI, MACE risk increased by 14% in patients >64 years [odds ratio (OR) 1.14 (1.01, 1.29), *P *= .03], 15% in patients with BMI ≥24 kg/m^2^ [OR 1.15 (1.02, 1.30), *P *= .02], 18% in females [OR 1.18 (1.01, 1.38), *P *= .03] and 10% in hypertensive patients [OR 1.10 (1.01, 1.18), *P *= .02]. Interaction analysis showed no significant effect modification by these factors.

### Impact of time-dependent variables on MACE risk

To address the potential for bias introduced by events occurring during follow-up and to examine the influence of key time-varying clinical factors, we conducted analyses using time-dependent Cox proportional hazards models (Table 5). Specifically, we evaluated the impact of dynamic changes in hs-cTnI, BNP, hemoglobin and time on dialysis (all treated as time-dependent covariates) on the risk of MACE. As detailed in Table 5, after full adjustment, each 10 ng/L increase in the time-updated hs-cTnI value remained significantly associated with a 2.71% elevated risk of MACE [HR 1.0271 (1.0172, 1.0372)].

**Table 5:** Time-dependent Cox analysis of MACE.

	HR (95% CI)		
Variable	Model I	Model II	Model III
hs-cTnI for 10 ng/L↑	1.0272 (1.0177–1.0368)	1.0286 (1.0188–1.0384)	1.0271 (1.0172–1.0372)
BNP	1.0002 (1.0001–1.0003)	1.0002 (1.0001–1.0003)	1.0002 (1.0001–1.0003)
Hemoglobin	1.0201 (1.0106–1.0297)	1.0194 (1.0097–1.0291)	1.0199 (1.0102–1.0298)
Months on dialysis	0.9961 (0.9912–1.0011)	0.9956 (0.9905–1.0007)	0.9963 (0.991–1.0015)

Model I unadjusted.

Model II adjusted for: age, sex, type of dialysis vascular access.

Model III adjusted for: age, sex, type of dialysis vascular access, hypertension and diabetes mellitus.

CI, confidence interval.

### CIF curve

**Figure 5:**
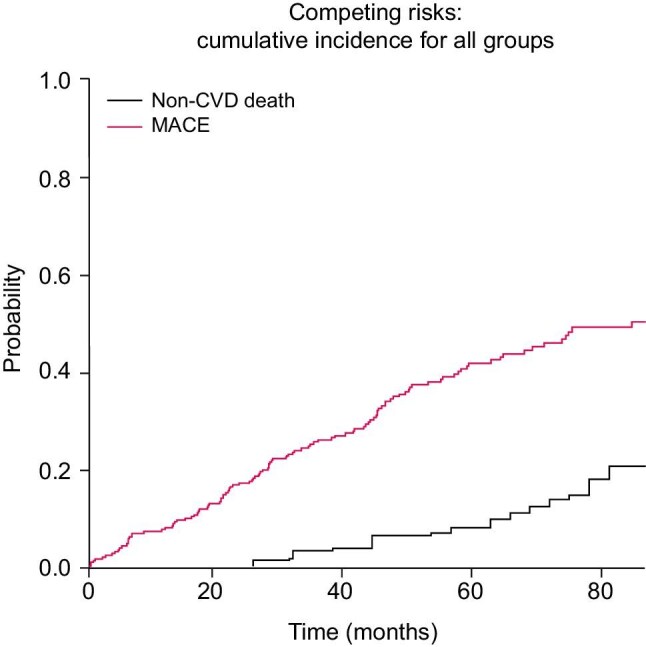
CIF curve for MACE and non-cardiovscular death with associated serial hs-cTnI trajectories.

To properly account for the high competing risk of non-cardiovascular death, which is particularly relevant in end-stage renal disease (ESRD), we performed analyses using the CIF. These analyses incorporated associated serial hs-cTnI trajectories as a time-dependent covariate. The resulting CIF curves demonstrated a significantly higher cumulative incidence of cardiovascular events compared with non-cardiovascular death across the cohort (Fig. 5).

### Relationship between hs-cTnI trajectory and MACE

**Figure 6:**
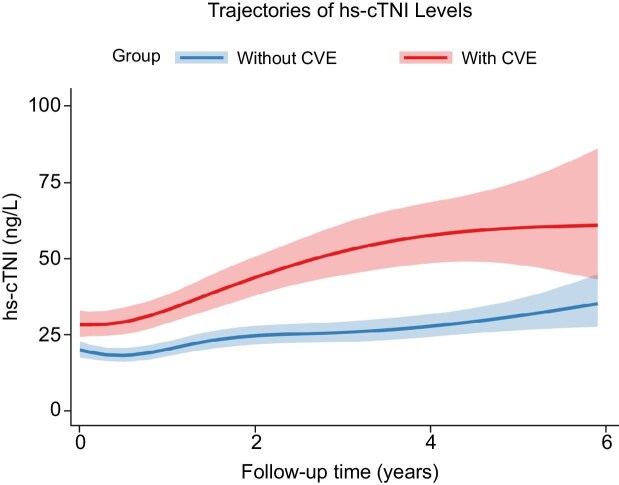
Temporal spline-based trajectories of hs-cTnI levels (ng/L) prior to MACE. The red line represents the trajectory of hs-cTnI (ng/L) in individuals with MACE; the blue line represents the trajectory of hs-cTnI (ng/L) in individuals without MACE.

The Time-Spline model, as illustrated in Fig. 6, was specifically developed to capture potential non-linear temporal dynamics in hs-cTnI. Compared with the non-MACE group, patients who experienced MACE exhibited a significantly steeper and progressively upward non-linear trajectory of hs-cTnI levels throughout the follow-up period. The hs-cTnI concentrations were not only consistently higher in the MACE group, but the absolute difference between the groups widened over time. To quantify the annualized rate of change within this non-linear framework, the average annual increase in hs-cTnI was estimated to be 0.34 ng/L [95% CI (0.14, 0.54), *P *< .001] in the MACE group, a rate which was significantly greater than that observed in the non-MACE group.

## DISCUSSION

In this retrospective cohort study of MHD patients, we observed a significant association between elevated hs-cTnI levels and the incidence of MACE. Over a 7-year follow-up, patients who experienced cardiovascular events exhibited distinct hs-cTnI trajectories, characterized by higher concentrations and a faster rate of increase compared with those without events.

In the general population, sustained or fluctuating elevations in troponin levels are strongly linked to increased mortality and cardiovascular risk [33–35]. Studies in general populations—such as Whitehall II, the Atherosclerosis Risk in Communities (ARIC) and the Trøndelag Health Study—have established the prognostic value of serial troponin measurements. For instance, the Whitehall II cohort demonstrated that individuals who died from cardiovascular disease exhibited a progressive increase in hs-cTnI over a 15-year period, years before the event [31]. Similarly, the Trøndelag Study showed a gradual rise in troponin preceding incident cardiovascular events [32]. The ARIC study further confirmed these patterns with hs-cTnT, linking 6-year changes to coronary heart disease, heart failure and mortality [33].

However, these foundational findings originate from cohorts with preserved renal function and cannot be directly extrapolated to the MHD population. This group is characterized by a unique cardiovascular risk profile, a high burden of chronic myocardial injury, and altered troponin metabolism and clearance. Consequently, the kinetics of hs-cTnI in MHD patients and their specific relationship with cardiovascular outcomes remain distinctly underexplored. Our study addresses this gap by providing one of the most detailed longitudinal analyses of hs-cTnI in an MHD cohort, with frequent sampling over 6 years. We move beyond population-based data to show that dynamic hs-cTnI trajectories offer critical prognostic information specific to the pathophysiology of heart disease in renal failure, thereby extending the paradigm of troponin monitoring into a high-risk population where its utility is both promising and poorly defined.

Data on dynamic troponin trajectories and their association with cardiovascular outcomes in hemodialysis patients remain limited. The work by Apple *et al*. established that elevated baseline cTnT and cTnI levels in patients undergoing chronic intermittent hemodialysis were associated with a 2- to 5-fold increase in mortality, noting a higher prevalence of cTnT elevation [11]. In contrast, Mavrakanas *et al*. reported that the majority of hemodialysis patients in their cohort did not exhibit elevated troponin levels at baseline; however, they found that persistently elevated or fluctuating cTnI levels over three consecutive monthly measurements were predictive of higher mortality and cardiovascular event rates at 1 year [35]. Our study builds upon this foundation by frequently measuring high-sensitivity troponin I (hs-cTnI) over an extended 6-year period, with an average of six measurements per patient. We not only confirm the prognostic value of elevated baseline hs-cTnI but also provide novel longitudinal evidence that patients who experienced MACE exhibited a significantly steeper upward trajectory in hs-cTnI over time compared with those who remained event-free. Furthermore, we observed that the absolute difference in hs-cTnI concentrations between these groups widened progressively throughout the follow-up. This strengthens the emerging hypothesis that serial troponin monitoring may offer superior sensitivity and higher predictive value for risk stratification than single-point measurements in the MHD population.

Stratified analysis highlighted that hs-cTnI is particularly useful for identifying high-risk subgroups, including the elderly, overweight individuals, women, and hypertensive patients. Evidence suggests that troponin levels increase linearly with age, as demonstrated in a cross-sectional study of 5764 community-dwelling elderly individuals [36]. Moreover, elevated hs-cTnI in older adults predicts cardiovascular mortality risk even before clinical diagnosis [37]. The relationship between BMI and troponin is more complex, with recent studies indicating gender-specific differences [38, 39]. For example, Kimenai *et al*. found that women had a 1.7-fold higher cardiovascular risk than men at a hs-cTnI concentration of 10 ng/L [40], potentially due to hormonal, vascular and immunological factors. These findings emphasize the need for gender-specific risk assessment protocols. In hypertensive patients, hs-cTnI has shown independent predictive value for cardiovascular events [41], supporting its utility in precision management strategies.

Our study has several strengths. First, we selected hs-cTnI as the primary biomarker due to its lower biological variability and greater reliability compared with hs-cTnT [42]. Second, our study reflects recent trends in cardiovascular morbidity among MHD patients, with a lower absolute number of events. Third, repeated hs-cTnI measurements (average of six per patient) improved the accuracy of cardiovascular risk prediction, enabling earlier identification of high-risk individuals for timely intervention.

However, this study has limitations. As a single-center retrospective study with a relatively small sample size (*n* = 265) and limited follow-up duration, our findings may not fully capture long-term outcomes. Future research should expand the sample size and conduct multicenter, prospective studies to validate the predictive stability of hs-cTnI in MHD patients. Additionally, exploring the combined use of hs-cTnI with other biomarkers and investigating treatment strategies for patients with elevated hs-cTnI levels will be critical for improving cardiovascular health management in this population.

Based on the above studies, it is recommended that the group variability of hs-cTnI test results be fully considered in clinical practice, and individualized interventions, such as more stringent blood pressure control regimens, should be implemented for patients with different risk profiles. It is important to emphasize that while most existing studies have focused on the general population with diverse risk factors, MHD patients remain an understudied group. Multicenter, high-quality prospective studies should prioritize exploring the predictive value of hs-cTnI in this specific population, as this could significantly enhance the cardiovascular risk management framework for patients with ESRD.

## CONCLUSION

In summary, hs-cTnI is a strong predictor of MACE in MHD patients, with those experiencing cardiovascular events showing a faster rise in hs-cTnI concentrations over time. Dynamic monitoring of hs-cTnI can enhance early risk identification and guide personalized interventions to improve cardiovascular outcomes in this high-risk population.

## Data Availability

The data underlying this article will be shared on reasonable request to the corresponding author.
